# The usefulness of CanAssist Breast over Ki67 in breast cancer recurrence risk assessment

**DOI:** 10.1002/cam4.6032

**Published:** 2023-05-28

**Authors:** Mallikarjuna Siraganahalli Eshwaraiah, Aparna Gunda, Govind Babu Kanakasetty, Manjiri M. Bakre

**Affiliations:** ^1^ OncoStem Diagnostics Private Limited Bangalore India; ^2^ HCG Cancer Centre Bangalore India

**Keywords:** CanAssist breast, IKWG, intermediate Ki67

## Abstract

**Background:**

Assessment of Ki67 by immunohistochemistry (IHC) has limited utility in clinical practice owing to analytical validity issues. According to International Ki67 Working Group (IKWG) guidelines, treatment should be guided by a prognostic test in patients expressing intermediate Ki67 range, >5%–<30%. The objective of the study is to compare the prognostic performance of CanAssist Breast (CAB) with that of Ki67 across various Ki67 prognostic groups.

**Methods:**

The cohort had 1701 patients. Various risk groups were compared for the distant relapse‐free interval (DRFi) derived from Kaplan–Meier survival analysis. As per IKWG, patients are categorized into three risk groups: low‐risk (<5%), intermediate risk (>5%–<30%), and high‐risk (>30%). CAB generates two risk groups, low and high risk based on a predefined cutoff.

**Results:**

In the total cohort, 76% of the patients were low risk (LR) by CAB as against 46% by Ki67 with a similar DRFi of 94%. In the node‐negative sub‐cohort, 87% were LR by CAB with a DRFi of 97% against 49% by Ki67 with a DRFi of 96%. In subgroups of patients with T1 or N1 or G2 tumors, Ki67‐based risk stratification was not significant while it was significant by CAB. In the intermediate Ki67 (>5%–<30%) category up to 89% (N0 sub‐cohort) were LR by CAB and the percentage of LR patients was 25% (*p* < 0.0001) higher compared to NPI or mAOL. In the low Ki67 (≤5%) group, up to 19% were segregated as high‐risk by CAB with 86% DRFi suggesting the requirement of chemotherapy in these low Ki67 patients.

**Conclusion:**

CAB provided superior prognostic information in various Ki67 subgroups, especially in the intermediate Ki67 group.

## Graphical abstract

Ki67 expression is often used to decide on use of chemotherapy. In a head‐to‐head comparison between Ki67 and CAB. CAB stratified 30% more patients as “low‐risk” (B) and thus spared more patients from chemotherapy compared to Ki67 based risk stratification (A). International Ki67 Working Group (IKWG) guidelines say, treatment should be guided by prognostic test in patients expressing intermediate Ki67 range of >5‐<30% in hormone receptor‐positive early breast cancer. CAB provided actionable prognostic information in intermediate Ki67 group (*n* = 655) as shown in C).

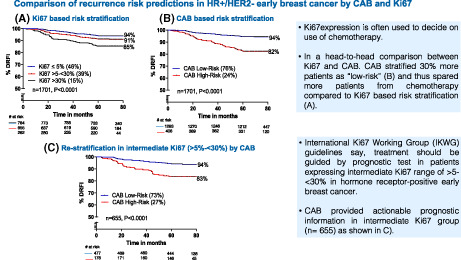

## 1 INTRODUCTION

Cellular proliferation in cancer cells is an index of the aggressiveness of tumor that suggests prognosis along with the prediction of the endocrine/chemotherapy benefit in breast cancer.^1^ The utility of Ki67 in early breast cancer is diverse as Ki67 is used in different scenarios like–an estimation of prognosis in response to adjuvant chemotherapy, chemotherapy benefit prediction, and to assess the response of a specific endocrine or chemotherapy regimen given in neoadjuvant settings.^2^ The most widely used method to measure Ki67 is immunohistochemistry.^3^ Current guidelines remain skeptical about the technical validity of the Ki67 by IHC as it is affected by multiple factors such as pre‐analytical, analytical factors, the IHC scoring method, and its interpretation. According to American Joint Committee on Cancer (AJCC) Ki67, a single factor could not be considered a reliable factor for its implementation in clinical practice to recommend a definite treatment protocol, because of the lack of reproducibility in the IHC technique,^4^, ^5^, ^6^ further compounded by the ambiguity due to various thresholds (on IHC gradings) used across various laboratories (14% and 20%) for making clinical decisions. According to the IKWG (International Ki67 in Breast Cancer Working Group) report, substantial interobserver and interlaboratory variability was observed in the ranges of >5% and < 30% with no such variations in Ki67 ranges of below 5% and above 30%.^7^ Thus, accommodating these variations in Ki67 IHC gradings, IKWG comments that the prognostic ability of Ki67 is limited and has reframed guidelines for the clinical utility of Ki67 in ER, PR positive HER2 negative stage I and II breast cancer.^2^ As per these guidelines, chemotherapy can be withheld in patients expressing <5% and patients expressing >30% should receive chemotherapy. Furthermore, IKWG recommends decisions on chemotherapy use for patients with Ki67 >5%–≤30% should be based on multiparameter gene expression prognostic assay results.^2^

Multi‐marker prognostic tests, Oncotype DX, MammaPrint, Prosigna, EndoPredict,^8^ are essentially gene‐based while CanAssist Breast (CAB)^9^ is a newer test that functions via protein biomarkers. Along with these multi‐marker prognostic tests, free online prognostic tests like NPI, PREDICT, and Modified Adjuvant Online (mAOL) are also used by clinicians to plan treatment in patients, especially in the limited resources geographies. CAB, is an IHC‐based test that analyses the expression of 5 protein (CD44, N‐Cadherin, pan‐Cadherin, ABCC4, and ABCC11) biomarkers. Clinical parameters (tumor size, Grade, and node status) along with IHC gradings are used by a machine learning‐based algorithm to compute a risk score denoting the probability of risk of distant recurrence.^9^ CAB segregates patients into two risk groups that is, low or high risk for recurrence. CAB has been validated on more than 3500 + patients across India, the USA, and Europe and has been used prospectively in 3000 + patients to date for treatment planning.^10^, ^11^, ^12^, ^13^

The objective of the current study is to compare the risk stratification of CAB with that of Ki67, as per these latest guidelines. The study has the following two main aims: (i) to compare the clinical outcomes of patients expressing low, intermediate, and high levels of Ki67 to that of CAB (low and high) risk groups, (ii) to assess and compare the performance of the CAB with online prognostic tools (NPI and mAOL) in the intermediate Ki67 range.

## 2 MATERIALS AND METHODS

### 2.1 Patient demographics

The current retrospective study is an analysis performed on archived postsurgically resected tumor tissues available as Formalin‐fixed paraffin‐embedded (FFPE) tumor tissues blocks from 1701 stage I, II, and IIIA hormone receptor (ER positive, PR positive or negative) positive, HER2/neu negative breast cancer patients that were obtained from hospitals and biorepositories from India, Europe (Spain, Italy, Austria, Germany), and USA diagnosed between 2007 and 2016. Clinical data of these patients along with age, date of diagnosis, treatment details, and clinical outcomes were obtained from the respective treating hospitals. Metastasis at a distant site within 5 years of breast cancer diagnosis is the study endpoint. Samples of patients who did not have an event at a distant site, with a minimum follow‐up of 5 years were only included in the study. Samples of patients treated with chemotherapy in neoadjuvant settings, samples of patients with incomplete clinical data or lack of information on clinical outcomes at 5 years since diagnosis or tumor samples that lack sufficient tumor content (as explained below) or improperly fixed were excluded from the study. The samples were received with the IRB and ethics committee approvals from all the participating hospitals and biorepositories. The data about the performance of CAB in this cohort (Indian and European cohorts) in different contexts have been analyzed and published previously.^12^, ^13^

**Quality check of tumor blocks:** The FFPE blocks received from the different hospitals and biorepositories were assessed for tumor quality by doing hematoxylin & eosin (H & E) staining.^9^ Tumor blocks with >30% tumor content and <20% necrotic content was processed further for Ki67 and CAB five biomarker IHCs.

### 2.2 Immunohistochemistry

**Ki67:** IHCs were performed at the OncoStem laboratory and graded by trained oncopathologists.^14^, ^15^ The Ki67 expression ≤5% was considered low risk, >5 < 30% intermediate, and ≥ 30% high risk.^2^

**CAB:** IHC was performed on five consecutive sections for CAB biomarkers on an automated Ventana platform.^16^ The IHC gradings along with node status, tumor size, and tumor grade were used as inputs into the proprietary algorithm to arrive at the risk category for each patient.^9^

### 2.3 NPI and adjuvant online

NPI uses the following equation entailing clinical parameters (tumor size, node, and grade) to arrive at an index.^17^

NPI = maximum invasive tumor size (S in cm) × 0.2 + lymph node stage (LN = 1, 2, or 3) + histological grade (H = 1, 2, or 3).

For the purpose of the current analysis, excellent and good prognostic groups were merged and called “Good Prognostic Group” (E + GPG) (NPI score ≤3.4); moderate I & II prognostic groups (NPI score >3.41 to ≤5.4) were merged and called “Moderate Prognostic Group” (MPG); and the poor prognostic group I & II (NPI score ≥5.4) are merged and called “Poor Prognostic Group” (PPG).

mAOL criteria as described in the MINDACT trial were used for assigning risk categories based on tumor grade, node status, and tumor size.^18^

### 2.4 Statistical analysis

Kaplan–Meier (KM) curves (GraphPad 8), and *p*‐values (Log‐rank test), were used to assess the association between CAB risk groups and clinical outcomes. DRFi (Distant Recurrence Free interval) for the risk groups was estimated from KM curves.

## 3 RESULTS

### 3.1 Study cohort

The study cohort included 1701 patients which comprise Indian (*n* = 782) and Caucasian (*n* = 919) patients. About 55% of the patients belonged to the age group, 41–60 (Table S1). 35% of the cohort belonged to stage I and 58% to stage II. 50% of the patients had T2 tumors and 61% and 63% had node‐negative and Grade 2 tumors respectively. 46% of the patients expressed Ki67 less than or equal to 5% (low‐risk) and 49% of the cohort expressed between the range >5%–<30% (intermediate‐risk). Only 15% of the population expressed Ki67 greater than or equal to 30% (high‐ risk). 55.4% of the patients were treated with chemotherapy. There were significant differences in age at diagnosis of breast cancer and tumor anatomical features between these Indian and Caucasian sub‐cohorts. The Indian cohort had significantly higher younger patients (>40 years) (12%) compared to the Caucasian cohort (4%, *p* < 0.0001) and significantly lower older patients (37%) than the Caucasian cohort (45%) (*p* = 0.0008). Patients with T2 tumors (70%) dominated in the Indian cohort as against T1 tumors in the Caucasian cohort (67.4%). Indian cohort had almost similar proportions of node‐negative (47%) and node‐positive (53%) patients while the Caucasian cohort predominantly comprised patients with node‐negative tumors (72%). Coming to tumor histological grades, the Indian cohort had a significantly lower number of patients with G1 tumors (10%) and more G3 tumors (31%) compared to Caucasians (G1‐15%; G3‐17%) cohort and these differences were statistically significant (*p* = 0.002 and <0.0001 for patients with G1 and G3 tumors respectively). Along with these differences in the tumor anatomical features, we also observed differences in Ki67 expression. While the Indian cohort had lower Ki67 expression (0.039), the Caucasian cohort had more patients expressing Ki67 in the intermediate range (*p* = 0.003) (Table S2). Lower‐stage, T1 tumors, N0 tumors, and G1 tumors were associated with low Ki67 and higher‐ stage, bigger tumors, N + tumors, and high‐grade tumors were associated with high Ki67 (Table S2).

### 3.2 Risk stratification by Ki67 and CAB

**FIGURE 1:**
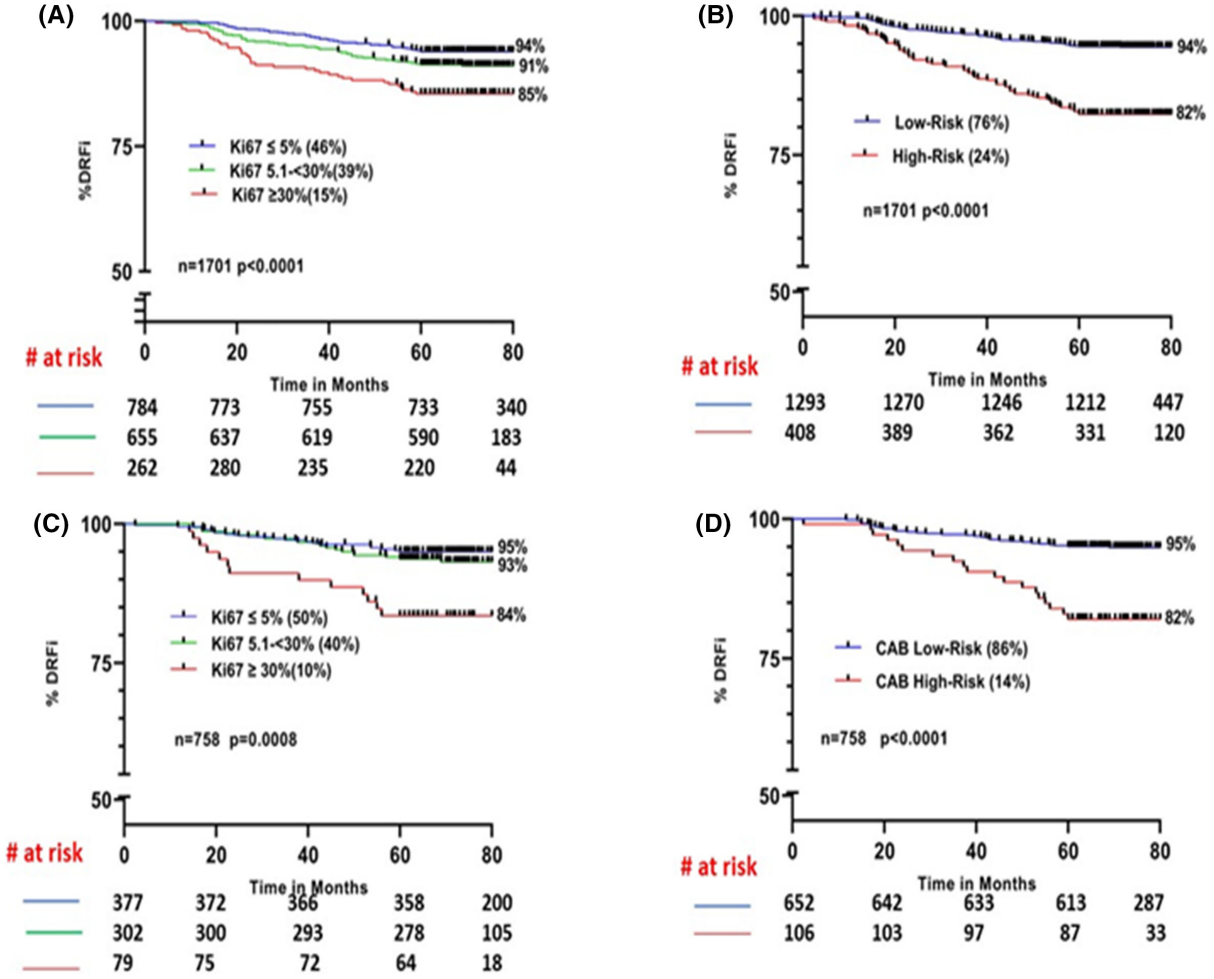
Kaplan–Meier survival curves for risk stratification by Ki67 and CAB: (A) Ki67 in total cohort (B) CAB in the total cohort (C) Ki67 in sub‐cohort of patients treated with endocrine therapy alone (D) CAB in sub‐cohort of patients treated with endocrine therapy alone.

In a head‐to‐head comparison between Ki67 and CAB, 46% of the patients were called low risk (≤5%) with a DRFi of 94%, 39% intermediate risk (>5%–<30%), and 15% high risk (≥30%) (*p* < 0.0001) (Figure 1A), whereas CAB stratified 76% as low‐risk with a DRFi of 94% (*p* < 0.0001) and 24% as high risk (Figure 1B). In stage, I and II patients (*n* = 1586), risk stratification by both was significant with a similar low‐risk DRFi (94%), but CAB stratified 32% more patients as low‐risk compared to Ki67 (Table 1).

**TABLE 1:** Risk stratification by CAB and Ki67 in various clinical sub‐groups.

Clinical sub‐group (total number of patients)	Prognostic test/marker	Low‐risk		Intermediate‐risk		High‐risk		*p*‐Value
		%	% DRFi	%	% DRFi	%	% DRFi	
Stage I + II (*n* = 1586)	CAB	78	94	NA		22	85	<0.0001
	Ki67	46	94	39	92	15	86	0.0002
Node‐negative (*n* = 1029)	CAB	87	96	NA		13	85	<0.0001
	Ki67	49	96	38	94	13	88	0.004
Node‐positive (*n* = 672)	CAB	59	92	NA		41	81	<0.0001
	Ki67	49	90	31	86	20	83	0.049
N1 (*n* = 585)	CAB	61	91	NA		39	85	0.015
	Ki67	41	92	39	88	20	84	0.059
T1 (*n* = 803)	CAB	84	96	NA		16	87	<0.0001
	Ki67	48	96	41	93	11	92	0.245
T2 (*n* = 844)	CAB	72	93	NA		28	80	<0.0001
	Ki67	44	92	37	89	19	82	0.0013
G1 (*n* = 224)	CAB	88	97	NA		12	85	0.002
	Ki67	59	98	32	93	9	90	0.057
G2 (*n* = 1073)	CAB	83	94	NA		17	82	<0.0001
	Ki67	45	93	40	92	15	89	0.167
G3 (*n* = 404)	CAB	51	83	NA		49	82	0.0007
	Ki67	42	92	37	89	21	79	0.0047

In patients treated with endocrine therapy (ET) alone (*n* = 758) with Ki67‐based risk stratification (Figure 1C), 50% of the patients were low risk (DRFi −95%) and 40% of patients belonged to the intermediate risk (DRFi‐93%) (*p* = 0.0008), whereas 86% were called LR by CAB with a DRFi of 95% (*p* < 0.0001) (Figure 1D).

### 3.3 Risk stratification in clinical sub‐groups

In a sub‐cohort of patients with lymph node‐negative disease, 49% expressed low Ki67 with a DRFi of 96% (*p* = 0.0044) (Table 1) while by CAB analysis 87% of patients were low‐risk with the same DRFi of 96% (*p* < 0.0001) (Table 1). In patients with lymph node‐positive tumors, we found that 41% were low‐risk by Ki67 while they were 59% by CAB with a DRFi of 90% in low Ki67 and 92% in CAB low‐risk (Table 1). In the analysis with N1 patients (up to 3 nodes positive), 41% expressed low Ki67 with a DRFi of 92% (*p* = 0.0595). In contrast, CAB stratified 61% of patients as low risk (Figure 1D) with a DRFi of 91% (*p* = 0.0157) (Table 1).

In the current cohort, 63% of the patients had Grade 2 tumors, 24% had Grade 3 tumors, and 13% had Grade 1 tumors (Table S1). In Grade 2 tumors Ki67 based stratification was not significant (*p* = 0.1671) (Table 1) whereas CAB was able to stratify 83% as LR and 17% as HR with statistical significance (*p* < 0.0001) (Table 1). In patients with Grade 3 tumors both Ki67 (*p* = 0.0047) and CAB‐based (*p* = 0.0007) stratification were significant (Table 1). In Grade 1 tumors Ki67 based stratification (Table 1) was marginally significant (p = 0.057) whereas CAB risk stratification was significant (*p* = 0.002) (Table 1).

In this cohort, the number of patients with T1 tumors (*n* = 803) and T2 (*n* = 844) tumors were similar (Table S1). In the T1 sub‐cohort Ki67‐based stratification was not significant (*p* = 0.2458) (Table 1) but CAB stratified patients significantly (*p* < 0.0001) into LR and HR groups with a DRFi of 96% and 87% respectively (Table 1). In the T2 tumor sub‐cohort both the Ki67 and CAB‐based stratifications were significant (*p* = 0.0013; *p* < 0.0001) (Table 1) with a similar DRFi in low‐risk (CAB LR DRFi = 93%; low Ki67 DRFi = 92% (Table 1).

### 3.4 Risk stratification by CAB in intermediate Ki67 sub‐cohort

**FIGURE 2:**
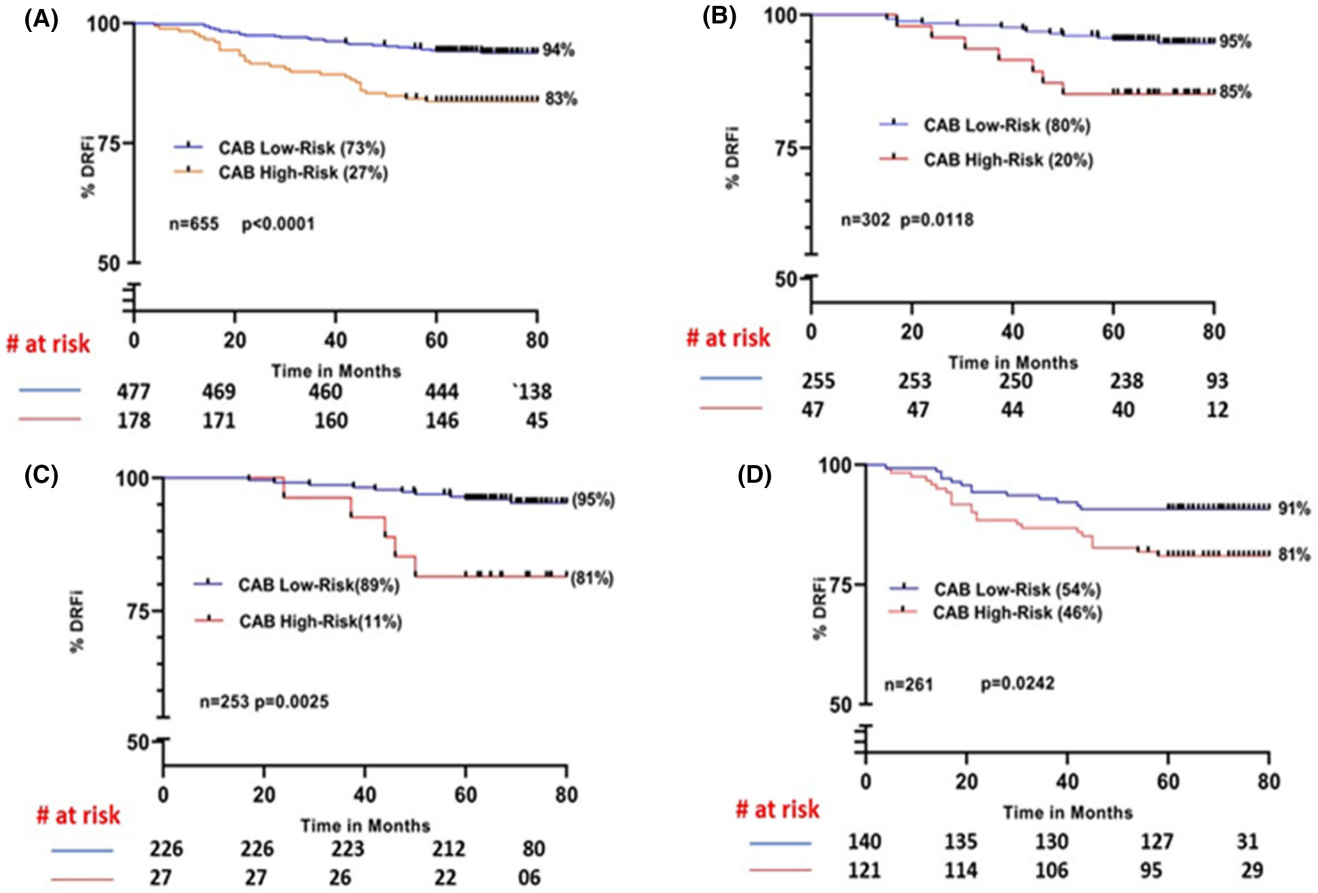
Kaplan–Meier survival curves for risk stratification by CAB in patients expressing intermediate Ki67 ranges (>5%–<30%): (A) total cohort (B) endocrine therapy alone sub‐cohort (C) node‐negative patients treated with endocrine therapy alone (D) node‐positive sub‐cohort.

In the sub‐cohort of patients expressing Ki67 in the intermediate zone (>5%–<30%) (*n* = 655), CAB was able to stratify 73% (*p* < 0.0001) of the patients into low‐risk with a DRFi of 94% (Figure 2A). In patients treated with endocrine therapy alone and expressing intermediate Ki67 levels (*n* = 302) CAB low and high‐risk groups were well segregated (*p* = 0.0118) with a low‐risk DRFi of 95% (Figure 2B). In the case of node‐negative sub‐cohort treated with endocrine therapy alone, 89% were low‐risk (*p* = 0.0025) with a DRFi of 95% (Figure 2C). Whereas in the node‐positive sub‐cohort, 54% (*p* = 0.0242) belonged to low‐risk with a DRFi of 91% (Figure 2D).

### 3.5 Comparison of CAB with NPI and mAOL in the intermediate Ki67 category

**FIGURE 3:**
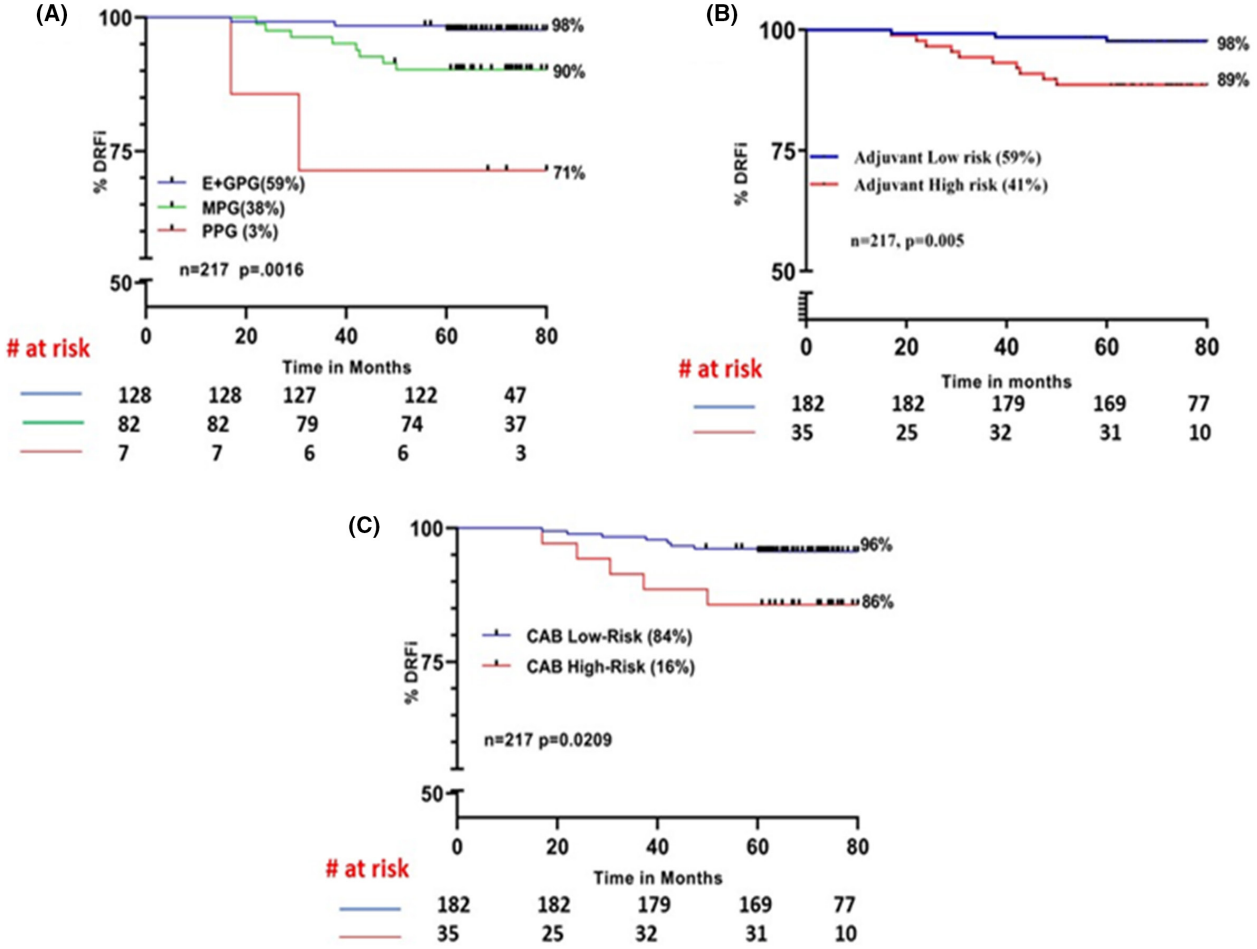
Kaplan–Meier survival curves comparing risk stratification by CAB and online prognostic tools in patients treated with endocrine therapy alone, expressing >5%–<30% Ki67 (intermediate Ki67 category): (A) NPI (B) Modified Adjuvant online (mAOL) (C) CAB

We computed the NPI score for 217 patients belonging to an intermediate Ki67 zone who were treated with endocrine therapy alone. In this sub‐cohort, both NPI and mAOL stratified 59% of patients as an excellent and good prognostic group/low risk with a 98% DRFi (Figure 3A,B). Whereas CAB in this cohort segregated 25% higher (*p* = 0.0025) patients compared to NPI and mAOL as LR. 84% of patients were LR by CAB with a DRFi of 96% (Figure 3C). Moreover, the 95% CIs of low‐risk or GPG groups for mAOL and NPI were slightly wider compared to CAB (Difference in upper and lower limit of 95% CI for NPI = 3.19, Adjuvant = 3.37, CAB = 2.02, data not shown), suggesting that prognostic information provided by CAB is superior to NPI and Adjuvant.

### 3.6 Risk stratification by CAB in low Ki67 (≤5%)

**FIGURE 4:**
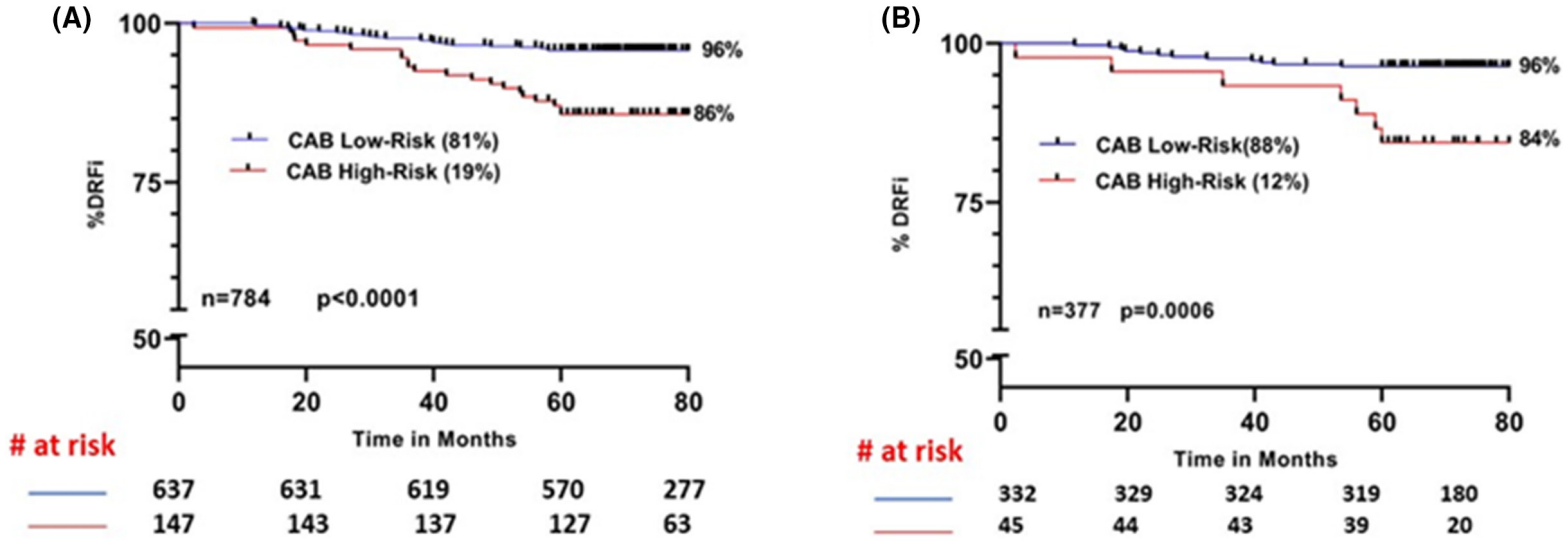
Kaplan–Meier survival curves for risk stratification by CAB in patients expressing low Ki67 (≤5%): (A) total cohort; (B) Patients treated with endocrine therapy (ET) alone.

We performed CAB‐based risk stratification in patients expressing low Ki67 in the total cohort and in patients treated with endocrine therapy alone (Figure 4). In both these sub‐cohorts, CAB identified 19% as high‐risk in the total cohort (Figure 4A) and 12% in the endocrine therapy alone sub‐cohort (Figure 4B) with a lower DRFi of 86% and 84% respectively. Moreover, CAB low‐risk patients expressing low Ki67 showed an improved DRFi of up to 96% (Figure 1A,B vs. Figure 4A,B).

### 3.7 Comparison of CAB and Ki67‐based risk stratification in cohorts of different ethnicities

Based on Ki67‐based risk stratification, in the Indian cohort (*n* = 782) 48% of the patients were stratified in the low‐risk group (Table 2) as against 44% of Caucasian cohort (Table 2) with a DRFi of 93% and 95% respectively. Based on CAB analysis, 74% in the Indian sub‐cohort, and 78% in the Caucasian sub‐cohort were stratified as low risk for recurrence with an identical DRFi of 94% in both cohorts (Table 2).

**TABLE 2:** Risk stratification by CAB and Ki67 in Indian and Caucasian patient cohorts.

Breast patient cohort	Prognostic test/marker	Low‐risk		Intermediate‐risk		High‐risk		*p*‐Value
		%	% DRFi	%	% DRFi	%	% DRFi	
Indian (*n* = 782)	CAB	74	94	NA		26	78	<0.0001
	Ki67	48	93	35	86	17	85	0.014
Caucasian (*n*‐919)	CAB	78	94	NA		22	86	<0.0001
	Ki67	44	95	42	93	14	85	0.049

## 4 DISCUSSION

Ki67‐based prognostication in clinical practice has its limitations due to analytical variability across laboratories. As per IKWG 2019 consensus, Ki67 ≤5% or ≥30% can be used to withhold or proceed with chemotherapy.^2^ Great variability for Ki67 IHC gradings ranging between 5% and 30%, was observed among investigators (*κ* = 0.6). Hence IKWG recommended the use of a prognostic test to plan therapy for patients with Ki67 in this range.^2^ The use of multi‐marker prognostic tests helped early‐stage hormone‐positive breast cancer patients avoid non‐beneficial chemotherapy.^19^ One such prognostic test is CAB which is an IHC‐based multi‐marker prognostic test. In the current study, we compared the risk stratification by CAB and Ki67 in different clinical subgroups with a special emphasis on the intermediate Ki67 group.

With CAB‐based risk stratification chemotherapy could be withheld in 30% more patients compared to Ki67 as CAB stratified 30% more patients as low‐risk in the total cohort and likewise across various clinical sub‐groups. Additionally in patients with small tumors (T1), and with commonly found histological Grade G2, and N1 tumors Ki67‐based risk stratification was not significant, while CAB risk stratification was significant; questioning the utility of Ki67 in these scenarios. It was earlier reported that the Ki67 biomarker as such lacks analytical validity and lacks evidence of good prognosis in node‐positive patients expressing low Ki67.^20^, ^21^, ^22^

The most important aspect of CAB in the context of Ki67 is its ability to stratify the patients expressing Ki67 in the intermediate range. Risk stratification analysis by CAB revealed that CAB stratified majority of these patients as LR who do not require to be treated with chemotherapy. In patients treated with endocrine therapy alone or patients with N0/N1 tumors expressing intermediate Ki67, up to 54% were identified as LR by CAB along with a 3% improvement in the DRFi. Moreover, in comparison to other online prognostic tools like NPI and ADJUVANT, it is worth to note CAB stratified 25% more patients as low‐risk compared to online/free tools along with a narrow 95% CIs for low‐risk DRFi for CAB compared to other two tools. Thus, this data establishes the higher utility of CAB over these online tools that primarily function through clinical parameters. This is in line with our data published recently in a much bigger cohort of ~1500 patients where we show CAB stratifies significantly more patients as LR compared to NPI.^23^

IKWG 2019 guidelines do not recommend chemotherapy in low Ki67 (≤5%). CAB extends its prognostic power in these patients with a low proliferative index once again in a very useful manner. While the low Ki67 group has more than 80% concordance with CAB low‐risk, CAB identified 19% of these patients as high‐risk that had a lower DRFi of 86%. This showcases the importance of CAB biomarkers in assessing the underlying aggressive tumor biology and identifying those who require to be treated with chemotherapy. To our surprise, there was a poor overall agreement between CAB and Ki67 (0–30% as low risk and above as high risk) with *κ* 0.07 (96% CI: 0.02–0.12). Many studies have shown a decent and significant overall concordance between Ki67 and Oncotype DX or MammaPrint.^24^, ^25^ However, like CAB, EPclin also failed to show a significant correlation with Ki67.^25^ The reason for such a concordance between Ki67 and Oncotype DX or MammaPrint could be that Ki67 is part of the regression equation that these two tests use, but Ki67 is not a part of either EPclin or CAB. The prognostic ability of CAB is majorly contributed by the critical biomarkers of various signaling pathways rather than the proliferation events.

The biology of breast cancer differs in cohorts from different ethnic backgrounds.^13^, ^26^ The Indian and Caucasian sub‐cohorts differed greatly with respect to clinical parameters. Even with these differences, the current study reports the similar performance of CAB in Caucasian and ethnic Indian cohorts, with respect to risk proportions (78%, 74%, *p* = 0.054) and low‐risk DRFi (94% in both cohorts) reiterating that CAB performance is beyond the influence of clinical parameters and racial/ethnic differences. However, with Ki67 we observed differences with respect to risk proportions across these two populations. While the Indian cohort had significantly lower intermediate Ki67 (35%) patients compared to the Caucasian cohort (42%; p = 0.0032), differences (albeit small) in DRFi in low and intermediate Ki67 groups were observed across both the cohorts with a similar DRFi in high Ki67 group and high Ki67 percentages across both the cohorts.

While these cutoffs are relatively new, we compared the CAB risk stratification with Ki67 at 14% and 20% cutoffs, (Table S3); an extension of our earlier study.^11^ At 14% cutoff, in the total cohort and in various sub‐groups (ET alone and N0), the percentage of CAB LR patients was significantly higher (*p* < 0.0001) than low Ki67 (<14%). Although with a 20% cutoff for Ki67, the risk stratification was similar to that of CAB; this data should be considered with caution in light of the current IKWG guidelines. Along with chemotherapy, another emerging therapy for high Ki67 patients is CDK4/6 inhibitors. These patients with high Ki67 can further be benefitted from abemaciclib as shown by monarchE trial results, which demonstrated an increasing absolute benefit of 6.4% with abemaciclib until 4 years.^27^ Risk stratification in these high Ki67 patients could be considered as an option to identify select patients who would derive maximum benefit of abemaciclib, and (CAB) high‐risk patients could be candidates for abemaciclib.^13^ In the current cohort with CAB restratification of high Ki67 (>20%) there is an increase in DRFi of 5% in CAB low‐risk patients compared to DRFi of all high Ki67 (>20%), suggesting the utility of enhanced therapy (abemaciclib) only to the CAB high‐risk patients (data not shown).

To our knowledge, this is the first report of its kind showing the usefulness of a prognostic test in the intermediate Ki67 range. The strength of the study is the large cohort size with patients from diverse ethnicities. The limitations are a lack of comparative analysis of the intermediate Ki67 range with other prognostic tests like multigene tests and the retrospective nature of the study. Although we have recently shown validation of long‐term predictions (10 years) of CAB^28^, in a DUTCH sub‐cohort of a prospective trial, TEAM, validation of CAB in a prospective trial per se is the limitation for CAB.

## 5 CONCLUSION

The clinical utility of Ki67 IHC in breast cancer care remains ambiguous because of the analytical variation and various thresholds used across laboratories. With CAB a large number of patients could withhold chemotherapy compared to Ki67‐based risk stratification. CAB demonstrated its usefulness over other online prognostic tools (NPI, ADJUVANT) in the intermediate Ki67 group, where guidelines indicate the use of a prognostic tool for clinical decision‐making. Thus, treatment decisions based on CAB in early ER+, HER2/neu negative breast cancer will prevent overtreatment in all patients and also in patients expressing low Ki67.

## AUTHOR CONTRIBUTIONS

**Mallikarjuna Siraganahalli Eshwaraiah:** Writing – original draft (equal). **Aparna Gunda:** Conceptualization (equal); data curation (equal); formal analysis (equal); writing – original draft (equal); writing – review and editing (equal). **Govind Babu K:** Conceptualization (equal); writing – review and editing (equal). **Manjiri M Bakre:** Funding acquisition (equal); writing – review and editing (equal).

## FUNDING INFORMATION

By private funding.

## CONFLICT OF INTEREST STATEMENT

Govind Babu has received fees for the advisory role from OncoStem Diagnostics in the past. All the rest of the authors are employees of OncoStem Diagnostics.

## Supporting information


**Table S1:** Patient demographics.
**Table S2**: Distribution of patients across different clinical subgroups in Ki67 prognostic groups.
**Table S3**: Risk stratification by Ki67 at 14% and 20% thresholds.

## Data Availability

Availability of data and materials: The data analyzed during the current study was not available publicly as it contains patient information. The data generated in the current work based on patient information will be available from the corresponding author upon request.
