# Supplementary material for: The usefulness of CanAssist Breast over Ki67 in breast cancer recurrence risk assessment
Source: Cancer Med. 2023 May 28;12(12):13342–51. doi: 10.1002/cam4.6032 (PMC10315758; doi:10.1002/cam4.6032)
Supplement: Supplementary file 1 — Table S1: Patient demographics. Table S2: Distribution of patients across different clinical subgroups in Ki67 prognostic groups. Table S3: Risk stratification by Ki67 at 14% and 20% thresholds. [file CAM4-12-13342-s001.docx]

**Supplementary Table 1:**

|  | **Total (n=1701)** | **Indian (n=782)** | **Caucasian (n=919)** |
| --- | --- | --- | --- |
| **Age** |  |  |  |
| < 40 | 133 (7.8%) | 92 (12%) | 41 (4%) |
| 41-60 | 933 (55%) | 465 (60%) | 468 (50%) |
| > 60 | 627 (37%) | 217 (27.6%) | 410 (45%) |
| unknown | 8 | 8 (1%) | 0 |
| **Stage** |  |  |  |
| Stage I | 590(35%) | 111(14%) | 479(52%) |
| Stage II | 996(58%) | 557(71%) | 439(48%) |
| Stage III | 114(7%) | 113(14%) | 1 |
| **T size** |  |  |  |
| T1 | 803 (47%) | 183 (23.4%) | 620 (67.4%) |
| T2 | 844 (50%) | 547 (70%) | 297 (32.3%) |
| T3 | 54 (3%) | 51 (6%) | 2 |
| T4 | 1 | 1 | 0 |
| **Node status** |  |  |  |
| N0 | 1029 (61%) | 368 (47%) | 661 (72%) |
| N1 | 585 (35%) | 327 (42%) | 258 (28%) |
| N2 | 78 (4%) | 78 (9%) | 0 |
| N3 | 9 | 9 (1%) | 0 |
| **Histological Grade** |  |  |  |
| G1 | 225 (13%) | 81(10%) | 144 (15%) |
| G2 | 1073 (63%) | 458 (58.5%) | 614 (66%) |
| G3 | 404 (24%) | 243 (31%) | 161 (17%) |
| **Receptor status** |  |  |  |
| ER Positive | 1682 (99%) | 766 (98%) | 917 (99.7%) |
| ER Negative | 18 (1%) | 15 (2%) | 2 |
| ER unknown | 1 | 1 | 0 |
| PR Positive | 1492 (88%) | 645 (82.4%) | 847 (92%) |
| PR Negative | 208 (12%) | 137 (17.5%) | 71 (7.7%) |
| PR unknown | 1 | 0 | 1 |
| **Chemo endocrine treated** | 943 (55.4%) | 627 (80%) | 316 (34%) |
| **Endocrine alone** | 758 (44.5%) | 155 (20%) | 603 (66%) |

**Supplementary Table 2:** Distribution of patients across different clinical subgroups in Ki67 prognostic groups

|  | **Ki67 low**  **(n = 784)** | **Ki67 intermediate**  **(n = 655)** | **Ki67 high**  **(n = 262)** |
| --- | --- | --- | --- |
| **Age** |  |  |  |
| <50 years | 247(44%) | 217(39%) | 98(17%) |
| >50 years | 533(47%) | 437(39%) | 161(14%) |
| **Race/ethnicity** |  |  |  |
| Indian | 380 (49%) | 271(35%) | 131 (16%) |
| Caucasian | 404 (44%) | 384 (42%) | 131 (14%) |
| **Stage** |  |  |  |
| Stage I | 290 (49%) | 244(41%) | 56(10%) |
| Stage II | 441(44%) | 369(37%) | 186(19%) |
| Stage III | 53(46%) | 41(36%) | 20(18%) |
| **T size** |  |  |  |
| T1 | 386(48%) | 328(41%) | 89 (11%) |
| T2 | 372(44%) | 310(37%) | 162(19%) |
| T3 | 26(49%) | 16(30%) | 11(21%) |
| **Node status** |  |  |  |
| N0 | 506(49%) | 394(38%) | 129(13%) |
| N1 | 237(41%) | 229(39%) | 119(20%) |
| N2 | 36(46%) | 29(37%) | 13(17%) |
| **Histological Grade** |  |  |  |
| G1 | 132(59%) | 72(32%) | 20(9%) |
| G2 | 479(45%) | 435(40%) | 158(15%) |
| G3 | 172(43%) | 148(36%) | 84(21%) |
| **Chemo Endocrine treated** | 407(43%) | 353(38%) | 183(19%) |
| **Endocrine alone** | 377(50%) | 302(40%) | 79(10%) |

Supplementary Table 3: Risk stratification by Ki67 at 14% and 20% thresholds

| Ki67 threshold | Cohort description | Low-risk | | High-risk | | P-value |
| --- | --- | --- | --- | --- | --- | --- |
|  |  | % | % DRFi | % | % DRFi |  |
| 14% | Total cohort (all patients) (n=1701) | 63 | 94 | 37 | 87 | <0.0001 |
|  | ET alone (patients treated with ET alone) (n=758) | 72 | 95 | 28 | 87 | <0.0001 |
|  | Node-negative (n=1029) | 68 | 96 | 32 | 90 | 0.0005 |
|  | Node-positive (n=672) | 57 | 91 | 43 | 83 | 0.0036 |
| 20% | Total cohort (all patients) (n=1701) | 78 | 93 | 22 | 84 | <0.0001 |
|  | ET alone (patients treated with ET alone) (n=758) | 84 | 95 | 16 | 83 | <0.0001 |
|  | Node-negative (n=1029) | 82 | 96 | 18 | 88 | <0.0001 |
|  | Node-positive (n=672) | 71 | 90 | 29 | 81 | 0.0007 |
